# Picturing Organisms and Their Environments: Interaction, Transaction, and Constitution Loops

**DOI:** 10.3389/fpsyg.2020.01912

**Published:** 2020-07-30

**Authors:** Ezequiel A. Di Paolo

**Affiliations:** ^1^Ikerbasque, Basque Foundation for Science, Bilbao, Spain; ^2^Centre for Computational Neuroscience and Robotics, University of Sussex, Brighton, United Kingdom; ^3^IAS-Research, University of the Basque Country, San Sebastián, Spain

**Keywords:** organism–environment relation, diagrams, schematic representation, interaction, transaction, constitution, enaction, ecological psychology

## Abstract

Changing conceptions of the relation between organisms and their environments make up a crucial chapter in the history of psychology. This may be approached by a comparative study of how schematic diagrams portray this relation. Diagrams drive the communication and the teaching of ideas, the sedimentation of epistemic norms and methods of analysis, and in some cases the articulation of novel concepts through pictographic variants. Through a sampling of schematic representations, I offer a concise comparison of how different authors, with different interests and motivations, have portrayed important aspects of the organism–environment relation. I compare example diagrams according to the features they underscore (or omit) and group them into classes that emphasize interaction, transaction, and constitution loops.

## Introduction

There are important convergences between ecological psychology and enaction but also differences. Some differences are due to historical accidents, as in the use of technical terms such as *information*. Enactivists are cautious about information–talk because they build their theory in opposition to notions of information traffic between agent and environment (although they do not reject the use of information-theoretic methods, e.g., Aguilera and Di Paolo, 2019; see also Beer and Williams, 2015). Ecological psychologists, in contrast, rely on a different concept of ecological information as regularities in the ambient array that help specify affordances and guide behavior (e.g., Reed, 1996). There are also differences in focus, with ecological psychology dealing traditionally with explanations of perception and perceptual development, and enaction typically more concerned with explanations of agency that do justice to human experience. Other differences are conceptual. Some of these revolve around ways of conceiving the relation between organisms and environments, conceptions that are rooted historically and not always spelled out.

In this article I look at a sampling of diagrams that express how different authors have conceived of the relation between organism and environment through the history of psychology. The exercise is limited but still helps to present a possible perspective according to which diagrams may be grouped according to the type of relation they underscore: interaction, transaction, and constitution loops.

Why look at diagrams instead of performing a well-documented textual analysis of the literature? Both are needed. But diagrams are powerful in driving the communication and the teaching of ideas. They help sediment perspectives and are one of the first tools used to approach new problems. Diagrams simplify; they select and they omit. What they leave out or distort is part of the narratives they help sustain (Tufte, 1997).

I am mostly concerned with schematic rather than realistic diagrams; pictorial simplifications that serve as conceptual anchors, what Rudolf Arnheim (1969) describes as “thinking with pure shapes.” They consist of simple elements: arrows conveying influence, lines and surfaces conveying boundaries, enclosed spaces conveying entities or processes, simple figures standing for objects, and short labels.

Diagrammatic thinking can lead to pictographic formalisms, as in the case of Feynman diagrams (Kaiser, 2005), Peirce’s existential graphs (Roberts, 1973), and bond graphs in engineering (Thoma, 1975). Most often, however, schematic diagrams occupy some point in between the normative sedimentation of ideas and the advance of novel thinking. Their productivity need not take the shape of a full-blown formalism and depends as much on the intellectual context as on the expressiveness of its conventions. Kurt Lewin’s topological diagrams in psychology^1^ (e.g., Lewin, 1936, 1938) show this, and so do [Bibr B28][Bibr B28]’s Isotype, and Moore (2016) extensions to the basic diagram of autopoiesis.

Some diagrams function as icons, others serve complex narratives and try to leave few aspects unaccounted. Many fulfill more than one function. Single depictions can afford close examination as in, for example, Evan Thompson’s analysis of Ernst Mach’s portrayal of his personal visual field (Thompson, 2007, pp. 280–82). Or a variety of illustrative diagrams can be put together to explore full theoretical frameworks, as in Turvey and Carello’s (1986) pictorial essay on ecological psychology. Here, I want to focus on single diagrams in relation with each other in order to uncover broad patterns and the ideas they convey.

The scope of this perspective is limited^2^ and the choice of examples and groupings follows my interest in highlighting three kinds of organism-environment relations: interaction, transaction, and constitution loops. These terms are described below. They are not meant as a novel categorization but as a way of looking at differences in emphasis. And of course, a diagram indicating relations of one of these types does not imply that its author is unconcerned by relations of the other types. The idea is to cautiously explore what diagrams suggest. The same material may be interpreted through alternative lenses, e.g., the kind and complexity of the pictographic conventions, the aesthetic dimension, or whether the emphasis is on structures or on processes, to mention a few possibilities.

## Interaction Loops

**FIGURE 1:**
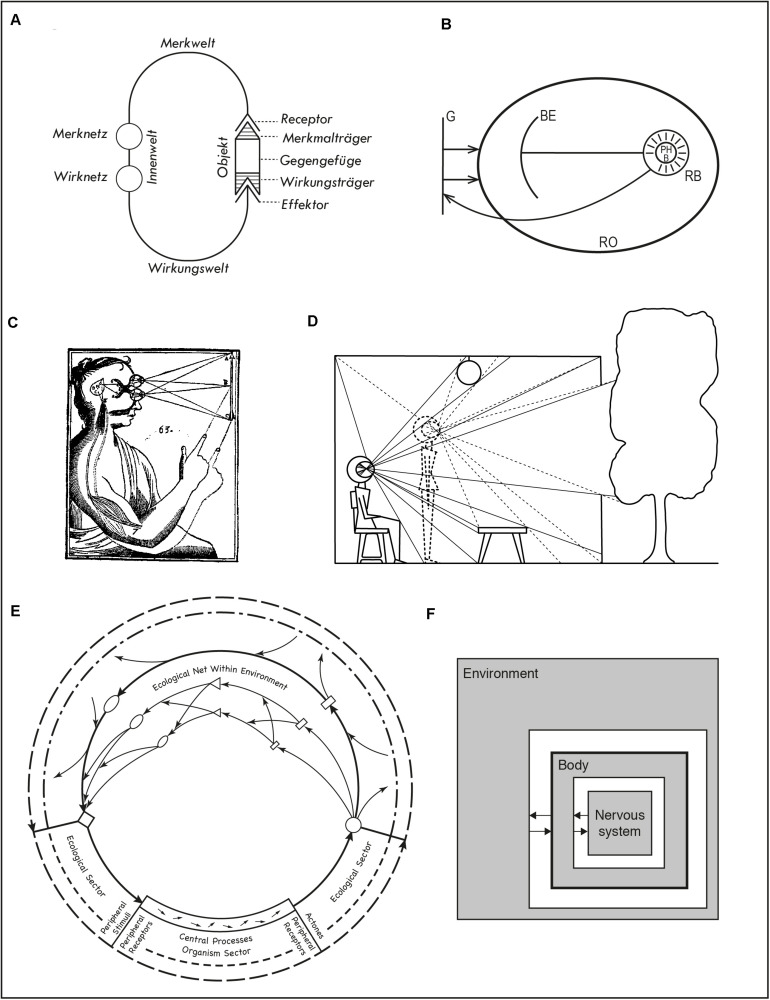
**(A)** von Uexküll’s functional circle. **(B)** Koffka’s depiction of the behavioral and geographical environment. **(C)** Descartes’ representation of a stimulation-action cycle. **(D)** Gibson’s depiction of lawful changes in the ambient array as a result of moving the observer. **(E)** Barker’s eco-behavioral circuits. **(F)** Beer’s iconic diagram of brain, body, and environment as coupled dynamical systems. See text for references.

**FIGURE 2:**
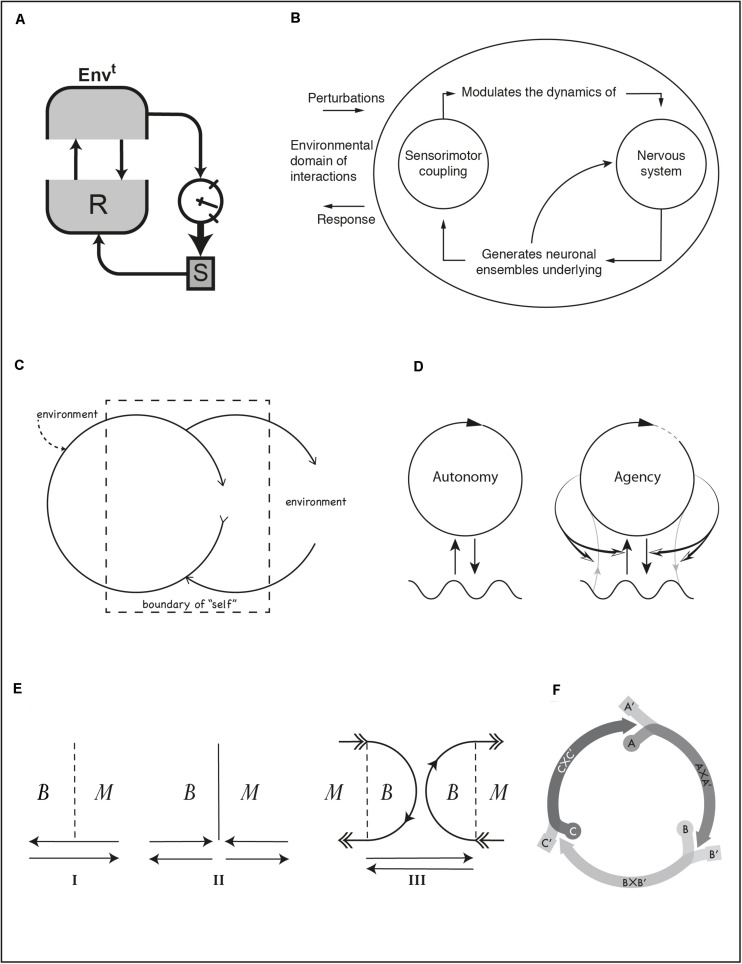
**(A)** Ashby’s ultrastable system. **(B)** Thompson’s depiction of internal processes in organisms with a nervous system. **(C)** Bateson’s conception of a self-constitution loop. **(D)** Iconic representation of a self-constituting autopoietic system **(left)** and enactive agent **(right)**. **(E)** Plessner’s distinction between nominal (I), reified (II), and processual (III) boundary between body and medium. **(F)** A sensorimotor scheme composed of three agent-environment coordination patterns. See text for references.

In almost every diagram that depicts organisms and their environments, we find arrows going from one to the other. Arrows convey influence and connection, and in most cases they form closed circuits to indicate that the relation between organism and environment is one of reciprocal influence. Closed loops are not a recent reaction to the classical “sandwich” model of the mind (Hurley, 1998). Analogous criticisms have been raised against simple stimulus-response thinking since the end of the 19th century (e.g., Dewey, 1896). We see loops depicted explicitly or implied in all of the diagrams in Figures 1, 2. Having said that, it is important to remind ourselves that open-loop explanations still abound in cognitive psychology and cognitive neuroscience.

Formally, an *interaction* is a mutual coupling between two dynamical systems. A system is coupled to another when its parameters and constraints depend on the state of the other system. The coupling is mutual if the same situation obtains in both directions. The *environment* of any given system is defined in dynamical terms as the set of all external variables to which the system is coupled and the sets of all external parameters it influences. Crucially, while the states of coupled systems change during interaction, the sets of variables, parameters, and formal relations do not change.

An important antecedent for both enaction and ecological psychology that describes this situation is Jakob von Uexküll’s depiction of the functional circle of an organism (Figure 1A; Von Uexküll and Kriszat, 1934, p. 7). The diagram shows a circuit going from an organism’s receptor organs to its effectors and closed by an external object. The character of the perceived environment is organism-dependent and constitutive of its inner world (*Innenwelt*). It depends, in particular on what actions the organism is capable of performing and what it is sensitive to, respectively, its *Wirkungswelt* and *Merkwelt*, as well as on the possibilities afforded by the object (*Mekrmalträger* and *Wirkungsträger*). The diagram presents on a same plane objective and subjective aspects of action/perception and serves to buttress von Uexküll’s concept of the *Umwelt*, the surrounding world of an organism.

A different attempt to establish the relation between the objective and subjective aspects of behavior was introduced by Koffka (1935, p. 40; Figure 1B). The diagram lacks von Uexküll’s elegant simplicity. The geographical (objective) environment (*G*) affects the real organism (*RO*), within which a relation is established between real behavior (*RB*, feeding back to *G*), phenomenal behavior (*PHB*), and the behavioral environment (*BE*). Koffka intends to illustrate the structure of the life space but the diagram is imperfect. Kurt Lewin (1936, 77) criticized its confusing conventions, such as the relation between real behavior (shown as an area), which takes place within the behavioral environment (shown as a line), yet is depicted as separate from it. Koffka’s points may be valid, e.g., the fact that not all action and perception processes are phenomenally conscious. But condensing such complex ideas in a line drawing is difficult. Simpler diagrams, like von Uexküll’s, travel further at the risk of blurring nuances.

Simplicity here is meant conceptually. Figure 1C shows a well-known illustration from Descartes’ *Treatise on Man* (Descartes, 1998, 154). Despite the artistic portrayal of a human body, it counts as a simple diagram. One source of bodily movement is the stimulation of the sense organs, which in turn induces activity in the pineal gland; from there a flow of spirits to the muscles activate a motor reaction. This is illustrated by the two positions of the arm, by the lawful relation between object and retinal stimulation, and by the internal circuit from eyes to brain to muscles. Formally, the diagram is a less abstract version of von Uexküll’s functional circle (Figure 1A), yet the intended meaning is quite different: one supports a mechanistic view where the body, like an automaton, is activated through stimulation (and other sources of activity in the pineal gland); the other conveys an inescapable subjective dimension of perception.

Descartes diagram is visually similar to a famous picture that Gibson (1986, p. 72; see also Gibson, 1963) used to make yet another different point (Figure 1D). Gibson was interested in moving beyond the special case of the static perceiver. Motion of the observation point reveals structural properties in the ambient array that are absent in the static case, such as variations in solid angles, changes in occlusions, and so on. As the array changes, some features and relations remain invariant. We see two stages in the motion of the whole body, from sitting to standing. This and similar diagrams have been used extensively in ecological psychology, e.g., to highlight the enabling effects of developmental changes (e.g., Adolph and Hoch, 2019, p. 144). Unlike Figure 1C, the internal arc of the sensorimotor loop remains implicit, while complex visual relations within the environment are shown explicitly. Pictorially, the requirements of depicting a body situated in an everyday environment and the lawful effects of motion on sensation are jointly met by replacing the whole head by the cross-section of a disproportionately large eye where light rays are inverted (as in Descartes’ diagram).

Figures 1E,F lie at opposite ends of representational complexity. Figure 1F is a well-known, iconic diagram produced by Randall Beer (e.g., Beer and Chiel, 2008; Figure 1) describing the reciprocal coupling between organism and environment. As in other cases (e.g., Warren, 2006, p. 367), its purpose is to support the formulation of mathematical expressions functionally connecting variables in the agent and the environment. The environment is depicted as surrounding the whole agent. Unlike other versions of the same diagram, a thicker line has been drawn around the square indicating the body. This highlights a certain unity of the agent within which two interactive systems have been indicated, the nervous system and the (rest of the) body. Context here is important. Beer has been using diagrams like this since the early 1990s (e.g., Beer, 1992) to accentuate the dynamic nature of each of the shaded areas and the notion that in principle none of them determines what goes on in the others. This contrasts with mainstream notions of staged processing prevalent in cognitivist and connectionist approaches. It also contrasts with the view that the brain controls the body as a puppeteer does. Moreover, the diagram conveys a subtler point: the whole organism, not its nervous system, interacts with the environment. The nervous system is not directly coupled with the environment, but indirectly and always through the body. This makes all the difference if we conceive the body as a dynamical system and not merely as a signal transductor.

Another interaction loop is shown in Roger Barker’s diagram (Figure 1E). It comes from his theory of behavior settings (Barker, 1968, p. 139) and depicts an organism engaged in various eco-behavioral circuits. The organism appears at the bottom of the large circles and is divided into peripheral receptors and effectors and central processes, in a way reminiscent of von Uexküll’s *Merknetz* and *Wirknetz*. Unlike von Uexküll’s single object, Barker shows various complex processes in the environment: relations between agent and objects (small circles, diamonds, and rectangles) both at the proximal level (e.g., a behavior such as catching a ball in a ball game) and ecological level (e.g., the playing field, other players). This diagram is animated by a richness of interactions between objects and even the dynamic character of the organism is underlined by a series of small arrows. In terms of the proportion of the loop occupied by the agent, Barker’s and von Uexküll’s diagrams are almost opposites. For Barker, the organism occupies a short segment in much larger loops that include many environmental processes. This is a suitable representation of his contention that when accounting for what groups of people do in everyday life, the behavior setting is usually the strongest determinant.

All of these examples show interaction loops in the sense that they do not explicitly depict any permanent change in the organization or structure of the systems involved. Such possibilities are not disallowed, but they are not emphasized either.

## Transaction Loops

Interaction loops are well-defined if the systems are well-defined. We are often, however, interested in how systems change. Once we allow organisms and environments to change structurally as a result of their engagement, the notion of interactive coupling becomes fuzzy as systems undergo a history of transformations. Variables and parameters may appear or disappear, functional relations may change. Such a history is better described by the concept of *transaction* (e.g., Dewey and Bentley, 1949), a situation where labels are only provisional as relations and processes undergo transformation. In developmental psychology, transactional models stress “the plastic character of the environment and of the organism as an active participant in its own growth” (Sameroff, 2009, 8). If systems may change, how do they sustain their identity? Maturana and Varela (1987) propose a distinction between organization (a set of formal relations) and structure (an actual instantiation of those relations) and suggest that the criterion of sameness is the conservation of organization even when structure changes, a process they define as *structural coupling*. We can then define transaction loops as processes of structural coupling whereby an agent’s organization is maintained but structures in the agent and the environment undergo a history of mutually enabled changes.

Figure 2A is a depiction of an ultrastable system, a concept developed by Ashby (1960, p. 83). The environment (*Envt*) is in a two-way coupling with the behavior generating sub-system (*R*) of the organism. Two other elements are shown that also belong to the organism: a set of parameters (*S*) that modulate the dynamics of *R* and a “gauge” indicating the state of organism’s essential variables, i.e., variables that must be kept within viability bounds for the organism to survive. A secondary feedback circuit connects all the elements in the diagram. An arrow from the environment to the gauge shows the effect of environmental states on the essential variables. An arrow from the essential variables to *S* indicates the triggering conditions that lead to changing the behavior control parameters. If changes in *S* affect *R* in such a way that essential variables at the viability boundary return to a safe zone, the system will have adapted to a new situation. Through this double feedback the organism undergoes a history of adaptive changes, i.e., a series of transactions. While the secondary, transactional, feedback is not operating, the first feedback instantiates a simple interaction loop.

Transactional relations are sometimes conveyed by describing the classes of processes at play. A typical diagram used in the enactive literature is Figure 2B (Thompson, 2007, p. 47). Similar diagrams appear in ecological psychology (e.g., Gibson, 1963, p. 12). A rather absent environment may be regarded as this diagram’s fault (contrast with Figure 1E or with an extended version in Chemero, 2009, p. 153). This diagram expresses the circular relations between processes within an agent with a nervous system, something deemed applicable to any environmental situation. The environment is the blank background from which arrows emerge carrying perturbations to the agent and sink carrying its responses. Other versions of this diagram (e.g., Varela, 1984, p. 319) add some symmetry and show the environment as an additional circle on the left. But Figure 2B is interesting in a perhaps unintended way. Read critically, diagrams like this may demonstrate a lack of attention toward environmental processes (cf. Barker’s diagram). Read more charitably, we should notice a broken convention in the use of arrows. Shortcutting semiotic levels, they point toward the diagram’s own background and not to another graphic element on the same plane. We may take this to signify a sense of inescapable environmental immersion. That diagrams may be assessed critically for their omissions or charitably for their subtlety underscores their semantic openness. Interpretation can reveal meanings intended implicitly, but also unintended meanings from which we can nevertheless draw interesting implications.

Looked at closely, even a sensorimotor scheme can count as a transaction loop although it involves only a behavioral scale typically conceived as interactive. Each segment in Figure 2F (Di Paolo et al., 2017, p. 85) stands for a joint coordination between organism and environment. Each coordination leads to a bodily and environmental situation that gives rise to the next coordination in the cycle. Coordination patterns are labeled, following Piaget, as AxA′, BxB′, CxC′, where A, B, and C are the bodily supporting processes (e.g., breathing, suckling, and swallowing when a baby is drinking from a milk bottle) and A′, B′, and C′ the supporting environmental processes (e.g., air, bottle, milk). Each coordination induces a transformation of the organism-environment relation such that at its end, the next coordination starts as a result. Each coordination thus fulfills functional and structural roles, and this fulfillment results from a history of past and ongoing equilibration. Unlike other diagrams, we see pure relations between organism and environment (bands that converge into an arrow segment), without explicitly schematizing either.

## Constitution Loops

We may sometimes be concerned not just with the historical transformation of organism and environment but with their very production, the coemergence of an individual together with its associated milieu (Simondon, 2005). If this is an ongoing process, as enactivists sustain, the continued existence of the organism as an entity must be the result of relations of *constitution*, i.e., relations by which organisms and environments co-emerge. These loops will often have a transactional character, but not all transactions entail relations of constitution which include organizational and as well as structural changes.

The meaning of arrows and closed shapes in most diagrams is usually straightforward. Arrows go from an “entity” (a closed shape) toward another “entity” or toward a relation (another arrow) in the case of modulatory couplings. The autopoiesis diagram (Figure 2D, left; Maturana and Varela, 1987, 74) re-signifies this convention: an arrow closes on itself forming a closed shape to indicate an entity constituted by circular relations between processes. This dialectical synthesis of conventions for entities and relations (circles and arrows) describes a constitution loop. The diagram has been adapted and extended many times, e.g., to illustrate ideas of minimal, sensorimotor, and linguistic agency, and social interaction^3^ (Di Paolo et al., 2018, pp. 54, 68, 197; see also Moore, 2016). For the enactive concept of agency (Figure 2D, right; Di Paolo et al., 2018, p. 54) modulatory arrows have been added that go from the self-constituting organism toward the environmental *coupling*, not toward the environment. This secondary loop may be seen as a generalization of Ashby’s ultrastable system. Gray lines indicate material exchanges that constitute the organism. They can also undergo regulation by the agent. The circle is not fully closed to signal that the agent is constantly in the process of making itself also through its actions.

The convention of the self-encircling arrow to indicate a constitution loop has been used before by Gregory Bateson (Figure 2C; Ruesch and Bateson, 1951, pp. 187, 189). Formally, if we ignore the dashed lines, this diagram and the autopoiesis diagram are identical, the only differences being the horizontal orientation, the fact that the circle describing the organism (“an entity with a self-correcting causal circuit,” p. 186) does not fully close on itself, and the missing wavy line, replaced by the label “environment” on the right. What distinguishes Bateson’s diagram is a dashed rectangle conveying the idea that the personal sense of “self” often combines both organismic and environmental processes and that parts of the body may sometimes be felt as belonging outside ourselves (thus also labeled “environment,” although this may cause confusion) and parts of our “self” include processes in the body’s environment (e.g., wearing glasses).

The idea of a self-producing entity that is itself constituted by the way it relates to its medium, though perfectly conceivable in scientific terms, is difficult to picture. In Figure 2E, Helmuth Plessner presents a comparison between views of the relation between body and medium (Plessner, 2019, p. 183; originally published in 1928). Inset I indicates a nominal boundary between body and medium (dashed line); interaction arrows freely transverse it in both directions. Inset II illustrates the boundary as a reified barrier, suggesting a domain of constitution on the left and a domain of interactions on the right, an idea similar to the doctrine of non-intersecting domains in the theory of autopoiesis. Inset III illustrates two coupled process arcs of construction and disintegration out of which both body and medium reciprocally constitute and distinguish themselves. The organism as a whole is “only half of its life” and demands environmental “supplementation without which it would perish” (Plessner, 2019, p. 180), a fundamental tension between openness and separation. The dialectical situation is reminiscent of Simondon’s (2005) philosophy of individuation and the enactive conception of life (Di Paolo, 2018).

## Discussion

This brief excursion does not exhaust the lessons we could draw from a more detailed comparison of schematic diagrams in psychology. More points can be made; more diagrams can be discussed. But it does produce some insights.

Pictorial or formal resemblance does not ensure that diagrams are used to make similar points, as we have seen in comparing Descartes’ diagram with von Uexküll’s and Gibson’s. It seems legitimate to ask whether similarity of representation might not sometimes suggest tacit convergences that are neither avowed nor rejected. Perhaps Descartes would not have entirely dismissed the dynamic interpretations in von Uexküll’s diagram, perhaps it makes some sense to link Gibson’s depiction of the observer in motion with von Uexküll functional cycle more explicitly (see Baggs and Chemero, 2018). Comparing diagrams can suggest novel interpretations and bring implicit ideas into the open.

There is a conceptual and practical distinction between interaction, transaction, and constitution loops even if some diagrams may ambiguously belong in more than one category. Establishing the timescale of interest may help in determining whether a situation is best treated as interactional (e.g., behavior) or transactional (e.g., learning and development). But this is not the only difference. Transactions do not only occur at longer timescales, and even when they do, their effects can still make a difference in the here and now of action and perception (like jumps in skill). Constitution loops are meant to describe how organisms are themselves always individuated through processes that constantly create the distinction between organism and environment. Their diagrammatic representation in self-encircling arrows graphically transcends the entity/relation distinction.

We may tentatively suggest that one difference between ecological psychology and enaction is that the former focuses more intensively on interaction and transaction loops, and the latter on transaction and constitution loops. This is only approximate and there are bound to be counterexamples (as in Randall Beer’s case, who has worked on models of interaction as well as models to clarify ideas of transaction and constitution in autopoiesis and enaction, e.g., Beer, 2020). Nor is there any implication that the situation must stay like this. But the suggestion may act as common ground in discussing the differences between the two approaches as well as pointing to transaction loops as a fertile zone for collaborative work.

## Author Contributions

The author confirms being the sole contributor of this work and has approved it for publication.

## Conflict of Interest

The authors declare that the research was conducted in the absence of any commercial or financial relationships that could be construed as a potential conflict of interest.

## Footnotes

1 Lewin’s use of abstract diagrams is fundamental in the development of his dynamical approach to psychology and deserves more extensive treatment than we can provide here.

2 Due to space and format constraints, only a small sample of 12 representative diagrams is shown here. Other diagrams are mentioned briefly in the text. All diagrams with the exception of Figure 1C have been (re)drawn by the author with permission and following as closely as possible the original sources (including placement of elements and types). Figures 1A,C,E are taken from the public domain.

3 To clarify, *social* interactions, according to their operational definition in enactive terms (De Jaegher and Di Paolo, 2007), can and usually do comprise interaction, transaction, and constitution loops. They are not only interactive in the restricted sense used here even if, for reasons of continuity with social psychology and social science, they are labeled as social *interactions*.
